# Incidence and mortality rates of lip, oral cavity, and pharynx cancers in Brazil: time-trend and age-period-cohort analysis from the last 30 years, Global Burden of Disease Study

**DOI:** 10.1590/0037-8682-0286-2021

**Published:** 2022-01-28

**Authors:** Daniel Volpato Romagna, Max Moura de Oliveira, Lucas Guimarães Abreu, Caroline Stein, Fernando Neves Hugo, Renato Teixeira, Deborah Carvalho Malta, Mohsen Naghavi, Betine Pinto Moehlecke Iser

**Affiliations:** 1 Universidade do Sul de Santa Catarina, Faculdade de Medicina, Tubarão, SC, Brasil.; 2 Universidade Federal de Goiás, Instituto de Patologia Tropical e Saúde Pública, Departamento de Saúde Coletiva, Goiânia, GO, Brasil.; 3 Universidade Federal de Minas Gerais, Faculdade de Odontologia, Belo Horizonte, MG, Brasil.; 4 Universidade Federal do Rio Grande do Sul, Programa de Pós-Graduação em Epidemiologia, Porto Alegre, RS, Brasil.; 5 Universidade Federal do Rio Grande do Sul, Departamento de Odontologia Preventiva e Social, Porto Alegre, RS, Brasil.; 6 Universidade Federal de Minas Gerais. Belo Horizonte, MG, Brasil.; 7 University of Washington, Institute for Health Metrics and Evaluation, Seattle, WA, United States.; 8 Universidade do Sul de Santa Catarina, Programa de Pós-Graduação em Ciências da Saúde, Tubarão, SC, Brasil.

**Keywords:** Head and neck câncer, Epidemiology, Incidence, Mortality, Cohort effect, Global Burden of Disease

## Abstract

**INTRODUCTION:**

Cancers are the second main cause of morbidity worldwide, but robust information on lip, oral cavity, and pharynx cancers in Brazil is lacking. This study aimed to analyze the trends of incidence and mortality caused by lip, oral cavity, and pharynx cancers and age-period-cohort effects in the Brazilian population of 30 years of age and over, in the period of 1990 to 2019.

**METHODS:**

A time series study of the incidence and mortality rates for oral cavity and pharynx cancer (“Lip and oral cavity cancer”, “Nasopharynx cancer”, and “Other pharynx cancer”) was conducted, with corrected data from the Global Burden of Disease Study (GBD) 2019. Age-standardized rates per 100,000 inhabitants, for the global population, were gathered according to the individuals’ sex. The annual average percentage change (AAPC) was estimated, as was the age-period-cohort effects.

**RESULTS:**

The incidence and mortality rates were higher for men in the studied anatomical regions. The cancers tended to decrease for men, except for nasopharynx cancer, which increased in individuals of both sexes. Mortality tended to present a decline in most of the groups studied. For men and women, the age-period-cohort model presented a better adjustment for both incidence and mortality.

**CONCLUSIONS:**

Incidence and mortality caused by the main head and neck cancers showed a tendency to decline over the past 30 years in Brazil, except for nasopharynx cancer, which showed an increase in incidence and mortality in some segments of the population. Higher rates were found for lip and oral cavity cancers in men.

## INTRODUCTION

Cancers are among the main problems of public health at a global level. They are the first or second main cause of death in developed and developing countries and rank between third and tenth in some under-developed countries^1^. Their incidence and mortality rates have been of significant epidemiological importance, as cancers affect both young and old individuals. Estimates have increased due to population growth, and differences between low- and high-income countries regarding the distribution and prevalence of etiological factors may occur^2^. In 2018, according to estimates, there were 18 million new cases of cancer in the world, and 9.6 million deaths caused by the disease^1^. In Brazil, in the same year, cancers caused more than 224,000 deaths, according to the National Cancer Institute (INCA, in Portuguese). It is estimated that for each year of the 2020-2022 period, there will be 625,000 new cases of cancer in the country^3^.

The head and neck region is a common location for the development of primary cancers, and squamous cell carcinoma (the main histological type of tumor) is the 6th most common malignancy in the world. This group includes malignant tumors, which affect the oral and nasal cavities, paranasal sinuses, pharynx, larynx, or salivary glands^4^, with the oral cavity and the pharynx being the most often affected places. In the United States, it is estimated that cancers in these regions will account for more than 54,000 new cases and 10,000 deaths in 2021^5^. Men are affected more often than women, in a ratio that ranges from 2:1 to 4:1. Most of those tumors have a disfavorable prognosis, with evolution to death when diagnosed and treated late. This occurs because of the scarce symptomatology in its early stages or due to the delay in seeking out medical attention^6^ ^,^ ^7^.

Among the conditions related to an increased risk for the development of head and neck cancers, in addition to smoking and alcohol consumption, one can also include eating habits; oral hygiene; exposure to ultraviolet light; occupational exposure to toxic agents; viral infections, such as Human Papillomavirus (HPV), Epstein-Barr virus (EBV), Hepatitis C virus (HCV), and Human Immunodeficiency virus (HIV); immunodeficiencies; and genetic factors^8^ ^,^ ^9^. It is estimated that up to two fifths of the new cases of cancer could be avoided by eliminating or reducing exposure to known risk factors^1^.

In Brazil, one study, analyzing the time trends of the mortality rates due to mouth and pharynx cancer, between 2002 and 2013, revealed stability in the number of deaths by the disease and different patterns in prevalence rates, according to sex, region of the country, and anatomical location^10^. Considering the need to update this data and the availability of data standardized and corrected for the last 30 years by the Global Burden of Disease (GBD) study^11^, the present study seeks to analyze the trends of incidence and mortality for lip, oral cavity, and pharynx cancers, as well as report the age-period-cohort effects on these cancers in the Brazilian population, between 1990 and 2019.

## METHODS

This time-series ecological study used estimates of incidence and mortality for head and neck cancer among adults, aged 30 years and older, in Brazil, from 1990 to 2019, produced by the Global Burden of Diseases and Injuries Study 2019, from the Institute of Health Metrics and Evaluation (IHME)^12^. To that effect, this study selected the following cancers: “Lip and oral cavity cancer” (ICD cades C00-C08.9, D10.0-D10.5, D11-D11.9), “Nasopharynx cancer” (C11-C11.9, D10.6), and “Other pharynx cancer” (C09-C10.9, C12-C13.9, D10.7), which, as a group, are referred to as ‘Head and Neck cancers’^11^.

The GBD cancer estimation process begins with mortality. The sources of these data are vital registration systems (Mortality Information System - SIM, in Brazil) and cancer registries. The data reported were mapped to a list of underlying causes in the GBD causes of a hierarchy of death^13^. Uninformative causes of death codes (the "garbage codes") are redistributed among appropriate underlying causes of death^14^. The population-based cancer records (RCBP, in Portuguese) were the sources used to calculate the incidence estimates^15^. As they are not available for all Brazilian States, mortality data are used to expand data availability; thus, mortality data are transformed into incidence through modeled mortality-to-incidence ratios (MIRs). The MIR modeling is specific to each cancer cause, sex, age group, location, and year, and uses data from locations where incidence and mortality of the same year are available. This model includes a linear-step mixed-effects model with logit link functions, with age, sex, and the Healthcare Access and Quality Index as covariates. The results of this step are smoothed over space and time and adjusted through a spatiotemporal Gaussian process regression^13^. Death data were included in cancer-specific Cause of Death Ensemble models (CODEm), and adjusted to independently modeled all-cause mortality (CodCorrect)^13^. 

Brazilian ethical precepts and specific resolutions were followed. Data was used in an aggregated format, without the identification of individuals and with no damage to them. The report of this study conforms to the Guidelines for Accurate and Transparent Health Estimates Reporting (GATHER) statement^16^. The authors declare that there is no conflict of interest.

The standardized rates of incidence and mortality for the global population and by age group^13^ per 100,000 inhabitants were calculated by the direct method. Such rates were determined for the selected cancers and their group, by sex and standardized by age (30 years of age and older) and age groups (30 to 59 years of age and 60 years of age and above). 

The AAPC was calculated to identify trends of incidence and mortality, with a 95% confidence interval (CI). The AAPC is the weighted average of the angular coefficients of linear regression, with weights equal to the length of each segment in the entire interval. An increase or reduction in the tendency is statistically significant when different from zero (p<0.05). An analysis of tendency was performed by linear regression using the Joinpoint Regression Program, version 4.8.0.1^17^.

For the age-period-cohort model, the ages were grouped in five-year intervals (beginning with 30 to 34 years of age and finalizing with 80 years of age and older), totaling 11 age groups. The periods were grouped in six intervals of five years each (1990-1994, 1995-1999, 2000-2004, 2005-2009, 2010-2014, and 2015-2019). The birth cohorts were estimated by the difference between the period and age group, beginning in 1910 and ending in 1985, with a total of 16 cohorts. 

This model is applied because the temporal tendencies are usually subjected to effects related to the subject’s age, lack of diagnosis or death (period), and year of birth (cohort)^18^. The effects of age, period, and birth cohort on incidence and mortality, according to anatomical location and sex, were estimated by the Poisson regression model. The estimate of relative risk (RR=1) considered the average cohort of the number of cases as the main reference (more stable cohort). The models (age, age-drift, age-cohort, age-period, and age-period-cohort) were compared, and the models with the least deviance were selected. The significance level was set at 0.05. The calculations were made using the Epi (version 2.42) package of the R software (version 4.0.3).

## RESULTS

### Incidence

**FIGURE 1:**
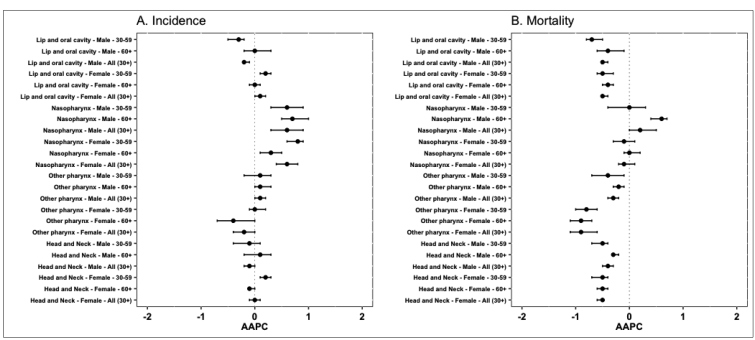
Annual Average Percentage Change (AAPC) and 95% Confidence Interval (CI) of the incidence and mortality rates of lip, oral cavity, and pharynx cancers, according to anatomical location, sex, and age group. Brazil, 1990-2019.

The age-standardized incidence rates for the group of cancers were 21.61 (1990) and 21.31 (2019) per 100,000 men, with a trend of decline [AAPC:-0.1 (95% CI:-0.2;-0.0)]; and of 5.33 (1990) and 5.34 (2019) per 100,000 women, with stability in the period. When analyzed according to the anatomical location and age group, the rates during the period were higher for men. Lip and oral cavity cancer showed the highest rates, with a tendency of decline for men of 30 to 59 years of age [AAPC: -0.3 (95% CI:-0.5;-0.2)] and for the total number of men (30 years of age and older) [AAPC: -0.2 (95% CI:-0.2;-0.1). Among women, there was an increase in the age group of 30 to 59 years [AAPC: 0.2 (95% CI:0.1;0.3)]. The lowest rates were verified for Nasopharynx cancer. However, a trend of increase for men and women was noticed. For cancers in other locations (other pharynx cancer), there was an increase for men over 60 years of age, and for the total number of men (30 years of age and older); and a reduction for women of 60 years of age and older and for the total number of women (30 years of age and older) (Figure 1 **,** Supplementary Material Table 1). 

**FIGURE 2:**
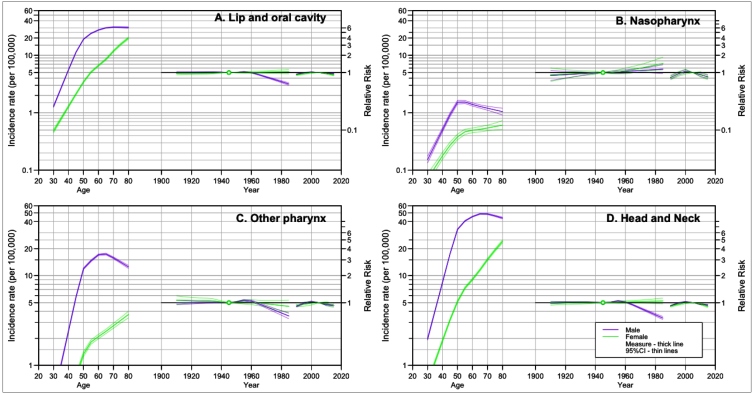
Age-Period-Cohort model for the incidence rate of lip, oral cavity and pharynx cancers, according to anatomical location and sex. Brazil, 1990-2019.

In the age-period-cohort models **(** Figure 2 **)**, incidence rates showed an increase for all age groups, in individuals from both sexes. Concerning the birth cohorts, when compared to men born in 1945, men born after 1965 showed a decline in risk, except for nasopharynx cancer. For female birth cohorts, no difference was observed. By period of diagnosis, when compared to 2005, there was a trend of decline in the most recent periods, for all anatomical locations and for individuals of both sexes (Table 1). For males and females, the complete model (age-period-cohort) was that which adjusted the best (p<0.001), except for Nasopharynx cancer and other pharynx cancers in women, for which the Age-Drift model showed a better adjustment (Supplementary Table 2). 

**TABLE 1:** Rate and Relative Risk and respective 95% CI of the incidence rate of lip, oral cavity, and pharynx cancers, according to anatomical location and sex. Brazil, 1990-2019.

Characterists		Incidence - Male				Incidence - Female			
		Lip and oral cavity cancer	Nasopharynx cancer	Other pharynx cancer	Head and Neck cancer	Lip and oral cavity cancer	Nasopharynx cancer	Other pharynx cancer	Head and Neck cancer
		Rate (95%CI)	Rate (95%CI)	Rate (95%CI)	Rate (95%CI)	Rate (95%CI)	Rate (95%CI)	Rate (95%CI)	Rate (95%CI)
Age group	30	1.28 (1.23;1.33)	0.15 (0.13;0.17)	0.43 (0.40;0.45)	1.96 (1.91;2.02)	0.48 (0.45;0.51)	0.07 (0.06;0.09)	0.11 (0.10;0.13)	0.64 (0.60;0.68)
	35	2.68 (2.60;2.76)	0.28 (0.25;0.31)	1.02 (0.98;1.06)	4.07 (3.98;4.16)	0.79 (0.74;0.83)	0.11 (0.10;0.13)	0.21 (0.19;0.23)	1.10 (1.05;1.15)
	40	5.60 (5.48;5.72)	0.51 (0.46;0.55)	2.42 (2.35;2.50)	8.43 (8.29;8.58)	1.29 (1.24;1.35)	0.18 (0.16;0.20)	0.40 (0.37;0.43)	1.89 (1.82;1.96)
	45	11.33 (11.12;11.55)	0.92 (0.85;1.00)	5.77 (5.63;5.91)	17.49 (17.24;17.74)	2.12 (2.05;2.20)	0.27 (0.24;0.31)	0.75 (0.70;0.80)	3.21 (3.11;3.32)
	50	19.04 (18.72;19.37)	1.53 (1.42;1.64)	11.95 (11.67;12.23)	32.74 (32.29;33.19)	3.43 (3.32;3.55)	0.38 (0.34;0.42)	1.34 (1.26;1.42)	5.15 (5.00;5.30)
	55	24.03 (23.64;24.43)	1.54 (1.45;1.63)	14.61 (14.28;14.96)	40.70 (40.18;41.23)	5.10 (4.95;5.25)	0.47 (0.42;0.52)	1.82 (1.72;1.92)	7.30 (7.11;7.49)
	60	27.59 (27.18;28.01)	1.39 (1.31;1.48)	17.08 (16.75;17.42)	45.48 (44.94;46.02)	6.64 (6.45;6.83)	0.50 (0.45;0.54)	2.10 (1.98;2.22)	9.09 (8.85;9.33)
	65	30.09 (29.60;30.59)	1.29 (1.20;1.38)	17.38 (17.01;17.76)	48.73 (48.10;49.36)	8.52 (8.26;8.79)	0.52 (0.46;0.57)	2.39 (2.27;2.53)	11.59 (11.30;11.89)
	70	30.95 (30.40;31.50)	1.20 (1.11;1.30)	15.77 (15.41;16.14)	48.54 (47.86;49.23)	11.84 (11.44;12.24)	0.54 (0.48;0.61)	2.77 (2.59;2.95)	15.09 (14.65;15.55)
	75	30.78 (30.14;31.42)	1.12 (1.01;1.24)	14.04 (13.63;14.46)	46.27 (45.51;47.05)	15.71 (15.15;16.30)	0.57 (0.50;0.66)	3.20 (2.98;3.42)	19.22 (18.62;19.84)
	80	30.44 (29.59;31.30)	1.05 (0.91;1.20)	12.49 (12.02;12.99)	43.79 (42.80;44.81)	19.92 (19.08;20.80)	0.61 (0.51;0.73)	3.68 (3.39;4.01)	24.05 (23.15;24.99)
		**RR (95%CI)**	**RR (95%CI)**	**RR (95%CI)**	**RR (95%CI)**	**RR (95%CI)**	**RR (95%CI)**	**RR (95%CI)**	**RR (95%CI)**
Cohort	1910	1.01 (0.97;1.05)	0.89 (0.73;1.09)	1.00 (0.95;1.06)	1.00 (0.97;1.03)	0.97 (0.92;1.02)	0.92 (0.70;1.20)	1.07 (0.96;1.19)	0.98 (0.94;1.03)
	1915	1.01 (0.98;1.04)	0.91 (0.77;1.07)	1.00 (0.96;1.05)	1.00 (0.98;1.03)	0.97 (0.93;1.01)	0.93 (0.75;1.16)	1.06 (0.97;1.16)	0.98 (0.95;1.02)
	1920	1.01 (0.99;1.04)	0.92 (0.80;1.06)	1.00 (0.97;1.04)	1.01 (0.99;1.03)	0.97 (0.93;1.00)	0.94 (0.79;1.13)	1.06 (0.99;1.14)	0.99 (0.96;1.02)
	1925	1.02 (1.00;1.04)	0.94 (0.84;1.04)	1.00 (0.98;1.03)	1.01 (0.99;1.03)	0.97 (0.94;1.00)	0.96 (0.84;1.10)	1.06 (0.99;1.13)	0.99 (0.96;1.02)
	1930	1.02 (1.00;1.04)	0.95 (0.88;1.03)	1.00 (0.98;1.03)	1.01 (1.00;1.02)	0.97 (0.94;1.00)	0.97 (0.88;1.08)	1.06 (0.99;1.12)	0.99 (0.97;1.02)
	1935	1.02 (1.01;1.04)	0.97 (0.93;1.01)	1.01 (0.99;1.02)	1.01 (1.00;1.02)	0.98 (0.95;1.00)	0.99 (0.91;1.07)	1.04 (0.99;1.10)	1.00 (0.97;1.02)
	1940	1.02 (1.01;1.03)	0.99 (0.96;1.01)	1.00 (0.99;1.02)	1.01 (1.00;1.02)	0.99 (0.98;1.01)	1.00 (0.94;1.06)	1.02 (0.99;1.04)	1.00 (0.99;1.01)
	1945	Reference	Reference	Reference	Reference	Reference	Reference	Reference	Reference
	1950	1.00 (1.00;1.01)	1.01 (0.97;1.05)	1.02 (1.01;1.03)	1.01 (1.01;1.02)	1.00 (0.98;1.02)	1.01 (0.96;1.06)	1.01 (0.98;1.03)	1.00 (0.99;1.02)
	1955	1.03 (1.02;1.05)	1.03 (0.9;1.09)	1.06 (1.04;1.08)	1.05 (1.04;1.06)	1.00 (0.98;1.03)	1.03 (0.95;1.11)	1.02 (0.97;1.08)	1.01 (0.98;1.04)
	1960	1.00 (0.98;1.02)	1.05 (0.98;1.12)	1.04 (1.02;1.07)	1.02 (1.00;1.03)	1.01 (0.98;1.04)	1.07 (0.95;1.20)	1.01 (0.95;1.08)	1.02 (0.98;1.05)
	1965	0.92 (0.90;0.94)	1.07 (0.99;1.15)	0.97 (0.95;1.00)	0.94 (0.93;0.96)	1.01 (0.98;1.05)	1.13 (0.99;1.28)	0.99 (0.93;1.06)	1.02 (0.99;1.06)
	1970	0.84 (0.82;0.86)	1.09 (1.00;1.18)	0.90 (0.87;0.93)	0.87 (0.85;0.88)	1.02 (0.97;1.07)	1.20 (1.04;1.38)	0.97 (0.89;1.06)	1.03 (0.99;1.07)
	1975	0.76 (0.74;0.79)	1.11 (0.99;1.23)	0.83 (0.79;0.87)	0.80 (0.78;0.82)	1.03 (0.97;1.09)	1.27 (1.07;1.51)	0.95 (0.85;1.06)	1.03 (0.98;1.08)
	1980	0.69 (0.67;0.72)	1.13 (0.98;1.30)	0.77 (0.72;0.81)	0.73 (0.71;0.76)	1.03 (0.96;1.11)	1.35 (1.10;1.67)	0.93 (0.81;1.06)	1.04 (0.98;1.10)
	1985	0.63 (0.60;0.66)	1.15 (0.96;1.37)	0.71 (0.66;0.76)	0.67 (0.65;0.70)	1.04 (0.95;1.13)	1.44 (1.12;1.86)	0.91 (0.77;1.07)	1.04 (0.97;1.12)
Period	1990	0.93 (0.92;0.95)	0.82 (0.77;0.87)	0.91 (0.89;0.93)	0.92 (0.91;0.93)	0.91 (0.89;0.93)	0.82 (0.75;0.90)	0.94 (0.90;0.99)	0.91 (0.89;0.93)
	1995	0.99 (0.98;1.01)	0.95 (0.90;1.01)	0.98 (0.96;1.00)	0.99 (0.98;1.00)	0.96 (0.93;0.98)	0.94 (0.86;1.03)	0.96 (0.91;1.01)	0.96 (0.94;0.98)
	2000	1.02 (1.01;1.04)	1.06 (0.99;1.12)	1.02 (1.01;1.04)	1.02 (1.01;1.03)	1.00 (0.97;1.02)	1.04 (0.94;1.15)	0.98 (0.94;1.03)	1.00 (0.97;1.02)
	2005	Reference	Reference	Reference	Reference	Reference	Reference	Reference	Reference
	2010	0.97 (0.95;0.98)	0.92 (0.86;0.98)	0.95 (0.93;0.97)	0.96 (0.95;0.97)	0.97 (0.94;1.00)	0.93 (0.84;1.03)	0.97 (0.92;1.02)	0.97 (0.95;0.99)
	2015	0.95 (0.94;0.97)	0.84 (0.79;0.90)	0.93 (0.91;0.95)	0.94 (0.93;0.95)	0.90 (0.88;0.93)	0.83 (0.75;0.92)	0.93 (0.89;0.98)	0.91 (0.88;0.93)

**RR:** Relative Risk; **CI:** Confidence Interval.

### Mortality

The age-standardized mortality rates for the head and neck cancers showed a decline from 16.42 (1990) to 14.71 (2019) per 100,000 men [AAPC: -0.4 (95% CI:-0.5;-0.3)]; and of 4.08 (1990) to 3.48 (2019) per 100,000 women [AAPC: -0.5 (95% CI:-0.6;-0.5)]. The trend of decline was also observed by age groups in individuals of both sexes. For lip and oral cavity cancer, the mortality rate showed a trend of decline in all the segments studied. For Nasopharynx cancer, although the rates were the lowest, a trend of increase was found for men aged 60 years and over and for the total number of men (30 years of age and older). For other locations (Other pharynx cancers), there was a trend of decline in mortality in the segments studied **(** Figure 1 **,** Supplementary Material Table 1). 

**FIGURE 3:**
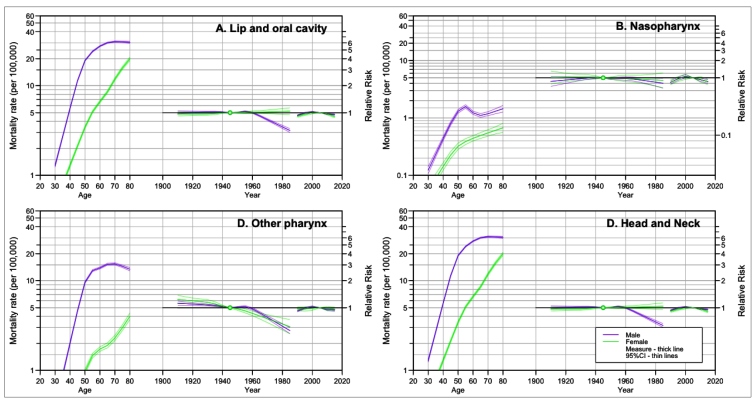
Age-Period-Cohort model for the mortality rate for lip, oral cavity, and pharynx cancers, according to anatomical location and sex. Brazil, 1990-2019.

For mortality, when the age-period-cohort models were analysed (Figure 3), an increase in mortality rates was noticed, for all of the age groups, and for individuals of both sexes. 

When compared to individuals born in 1945, a decline in risk was observed in more recent years, except for nasopharynx cancer in women. By period of diagnosis, a trend of decline was observed in more recent periods when compared to 2005 (Table 2). For both sexes, the complete model (age-period-cohort) was that which adjusted the best (p<0.001), except for other pharynx cancers in women, for which the Age-Drift model showed a better adjustment (p<0.001) (Supplementary Material Table 2). 

**TABLE 2:** Rate and Relative Risk and respective 95% CI of the mortality rate of lip, oral cavity and pharynx cancers, according to anatomical location and sex. Brazil, 1990-2019.

Characterists		Mortality - Male				Mortality - Female			
		Lip and oral cavity cancer	Nasopharynx cancer	Other pharynx cancer	Head and Neck cancer	Lip and oral cavity cancer	Nasopharynx cancer	Other pharynx cancer	Head and Neck cancer
		Rate (95%CI)	Rate (95%CI)	Rate (95%CI)	Rate (95%CI)	Rate (95%CI)	Rate (95%CI)	Rate (95%CI)	Rate (95%CI)
Age group	30	0.66 (0.63;0.70)	0.12 (0.11;0.14)	0.38 (0.36;0.41)	1.16 (1.11;1.20)	0.20 (0.18;0.22)	0.05 (0.04;0.07)	0.10 (0.08;0.11)	0.35 (0.32;0.37)
	35	1.35 (1.30;1.41)	0.23 (0.21;0.26)	0.88 (0.84;0.92)	2.46 (2.39;2.53)	0.34 (0.31;0.37)	0.09 (0.07;0.11)	0.17 (0.15;0.19)	0.59 (0.56;0.63)
	40	2.77 (2.69;2.86)	0.43 (0.40;0.47)	2.01 (1.94;2.08)	5.22 (5.10;5.33)	0.58 (0.54;0.61)	0.14 (0.12;0.17)	0.31 (0.28;0.34)	1.02 (0.98;1.07)
	45	5.67 (5.53;5.81)	0.80 (0.74;0.87)	4.61 (4.48;4.74)	11.08 (10.88;11.29)	0.98 (0.93;1.02)	0.23 (0.20;0.26)	0.55 (0.52;0.60)	1.76 (1.70;1.83)
	50	10.75 (10.50;11.01)	1.31 (1.22;1.40)	9.58 (9.33;9.84)	21.61 (21.24;21.99)	1.64 (1.58;1.71)	0.32 (0.28;0.36)	0.97 (0.91;1.03)	2.98 (2.89;3.08)
	55	14.50 (14.20;14.82)	1.58 (1.48;1.70)	12.93 (12.63;13.25)	28.87 (28.42;29.33)	2.62 (2.53;2.72)	0.39 (0.34;0.44)	1.46 (1.38;1.54)	4.55 (4.42;4.69)
	60	15.90 (15.55;16.26)	1.23 (1.15;1.31)	13.87 (13.53;14.22)	31.05 (30.55;31.56)	3.79 (3.67;3.92)	0.44 (0.40;0.49)	1.73 (1.63;1.83)	5.90 (5.73;6.06)
	65	17.43 (17.04;17.82)	1.11 (1.03;1.19)	15.25 (14.87;15.63)	33.79 (33.24;34.34)	4.88 (4.70;5.07)	0.49 (0.44;0.56)	1.90 (1.78;2.02)	7.15 (6.92;7.38)
	70	19.65 (19.17;20.14)	1.19 (1.10;1.28)	15.39 (14.97;15.82)	36.33 (35.68;37.00)	6.35 (6.08;6.63)	0.55 (0.48;0.62)	2.30 (2.15;2.46)	9.19 (8.88;9.50)
	75	22.47 (21.88;23.08)	1.31 (1.18;1.45)	14.46 (14.02;14.91)	38.42 (37.67;39.20)	9.69 (9.20;10.20)	0.61 (0.53;0.70)	2.99 (2.78;3.21)	13.42 (12.90;13.96)
	80	25.76 (24.89;26.66)	1.44 (1.27;1.65)	13.42 (12.88;14.00)	40.52 (39.48;41.59)	18.13 (17.22;19.08)	0.67 (0.56;0.81)	4.00 (3.68;4.36)	22.61 (21.68;23.58)
		**RR (95%CI)**	**RR (95%CI)**	**RR (95%CI)**	**RR (95%CI)**	**RR (95%CI)**	**RR (95%CI)**	**RR (95%CI)**	**RR (95%CI)**
Cohort	1910	1.10 (1.05;1.15)	0.87 (0.71;1.06)	1.12 (1.06;1.19)	1.10 (1.07;1.14)	1.10 (1.03;1.18)	1.01 (0.78;1.31)	1.23 (1.11;1.37)	1.12 (1.06;1.18)
	1915	1.10 (1.06;1.14)	0.89 (0.75;1.05)	1.11 (1.06;1.16)	1.10 (1.07;1.13)	1.08 (1.03;1.14)	1.02 (0.82;1.26)	1.21 (1.11;1.32)	1.11 (1.06;1.16)
	1920	1.09 (1.06;1.12)	0.92 (0.80;1.05)	1.10 (1.06;1.14)	1.09 (1.06;1.11)	1.07 (1.02;1.11)	1.02 (0.86;1.21)	1.19 (1.11;1.28)	1.10 (1.06;1.14)
	1925	1.09 (1.06;1.12)	0.94 (0.85;1.04)	1.08 (1.05;1.12)	1.08 (1.06;1.10)	1.05 (1.00;1.10)	1.02 (0.89;1.17)	1.17 (1.09;1.25)	1.08 (1.04;1.12)
	1930	1.08 (1.05;1.11)	0.97 (0.90;1.04)	1.07 (1.05;1.10)	1.07 (1.05;1.09)	1.04 (1.00;1.08)	1.03 (0.92;1.15)	1.15 (1.07;1.23)	1.07 (1.03;1.10)
	1935	1.06 (1.04;1.08)	0.99 (0.95;1.04)	1.06 (1.03;1.08)	1.06 (1.04;1.07)	1.03 (1.00;1.07)	1.03 (0.93;1.13)	1.10 (1.05;1.16)	1.05 (1.02;1.07)
	1940	1.02 (1.01;1.03)	1.01 (0.98;1.04)	1.03 (1.01;1.05)	1.03 (1.02;1.04)	1.02 (1.00;1.04)	1.02 (0.95;1.09)	1.04 (1.01;1.07)	1.02 (1.01;1.04)
	1945	Reference	Reference	Reference	Reference	Reference	Reference	Reference	Reference
	1950	1.02 (1.01;1.03)	0.97 (0.93;1.02)	1.02 (1.01;1.03)	1.01 (1.01;1.02)	0.97 (0.96;0.99)	0.99 (0.94;1.03)	0.97 (0.94;1.01)	0.97 (0.95;0.99)
	1955	1.01 (0.99;1.04)	0.97 (0.92;1.03)	1.03 (1.01;1.05)	1.02 (1.01;1.04)	0.94 (0.92;0.96)	0.99 (0.90;1.08)	0.92 (0.88;0.97)	0.94 (0.91;0.96)
	1960	0.95 (0.92;0.97)	0.97 (0.91;1.04)	0.97 (0.94;1.00)	0.96 (0.94;0.98)	0.91 (0.87;0.94)	0.98 (0.86;1.12)	0.86 (0.81;0.92)	0.90 (0.87;0.93)
	1965	0.84 (0.82;0.87)	0.95 (0.88;1.03)	0.87 (0.85;0.90)	0.87 (0.85;0.88)	0.88 (0.83;0.93)	0.97 (0.84;1.11)	0.81 (0.75;0.87)	0.86 (0.83;0.90)
	1970	0.75 (0.72;0.77)	0.91 (0.83;1.00)	0.78 (0.75;0.81)	0.78 (0.76;0.79)	0.85 (0.79;0.91)	0.95 (0.81;1.12)	0.75 (0.68;0.83)	0.83 (0.78;0.87)
	1975	0.66 (0.64;0.69)	0.88 (0.78;0.99)	0.70 (0.66;0.73)	0.69 (0.67;0.72)	0.82 (0.75;0.89)	0.93 (0.77;1.14)	0.70 (0.62;0.80)	0.79 (0.74;0.85)
	1980	0.59 (0.56;0.62)	0.84 (0.72;0.98)	0.62 (0.58;0.66)	0.62 (0.60;0.65)	0.79 (0.71;0.88)	0.92 (0.72;1.17)	0.66 (0.56;0.77)	0.76 (0.70;0.83)
	1985	0.52 (0.49;0.56)	0.81 (0.66;0.98)	0.56 (0.51;0.60)	0.56 (0.53;0.58)	0.76 (0.67;0.86)	0.90 (0.67;1.22)	0.61 (0.51;0.74)	0.73 (0.66;0.81)
Period	1990	0.94 (0.93;0.96)	0.83 (0.78;0.88)	0.92 (0.90;0.94)	0.93 (0.91;0.94)	0.94 (0.91;0.97)	0.84 (0.76;0.92)	0.97 (0.92;1.02)	0.94 (0.91;0.96)
	1995	0.99 (0.97;1.01)	0.96 (0.90;1.02)	0.99 (0.97;1.01)	0.99 (0.97;1.00)	0.97 (0.93;1.00)	0.95 (0.86;1.05)	0.97 (0.92;1.02)	0.97 (0.94;1.00)
	2000	1.02 (1.00;1.04)	1.06 (1.00;1.14)	1.03 (1.01;1.05)	1.02 (1.01;1.03)	0.99 (0.96;1.02)	1.04 (0.94;1.16)	0.98 (0.93;1.03)	0.99 (0.97;1.02)
	2005	Reference	Reference	Reference	Reference	Reference	Reference	Reference	Reference
	2010	0.97 (0.95;0.99)	0.93 (0.86;0.99)	0.95 (0.93;0.97)	0.96 (0.95;0.97)	0.98 (0.95;1.01)	0.94 (0.84;1.05)	0.98 (0.92;1.04)	0.98 (0.95;1.01)
	2015	0.96 (0.94;0.98)	0.86 (0.80;0.92)	0.94 (0.92;0.96)	0.95 (0.93;0.96)	0.93 (0.89;0.96)	0.85 (0.76;0.95)	0.96 (0.91;1.01)	0.93 (0.91;0.96)

**RR:** Relative Risk; **CI:** Confidence Interval.

## DISCUSSION

This study aimed to evaluate the most recent estimate of the time trend of the incidence and mortality rates of the main head and neck cancers, broken down by anatomical location in Brazil, from 1990 to 2019, using corrected data from the GBD. Lip and oral cavity cancer, in addition to being the most common in the studied population, showed the highest rates of mortality. Males had the highest rates of incidence and mortality for cancers of all of the anatomical locations. A trend of decline in incidence was noticed for males, except for nasopharynx cancer, which, although it showed the lowest rates, showed an increase in individuals of both sexes. There was a trend of decline in mortality, but not in all of the anatomical locations, and some difference was identified between male and female individuals. 

The highest incidence and mortality rates were found for men, for all the anatomical locations, which may well be related to the fact that, historically, men are more exposed to the risk factors that favor the development of cancer - smoking and alcohol consumption^19^ ^,^ ^20^ as well as excessive exposure to ultraviolet radiation^21^. However, historical trends have shown a reduction in smoking, which was more accentuated in men^19^ ^,^ ^22^. This finding may at least partially justify the reduction in the incidence of the disease, also affecting mortality. By contrast, an increase in alcohol consumption had been observed among individuals of 30 to 69 years of age^23^ as well as among women^19^ ^,^ ^24^, which might have impacted the stability of incidence in females. Nonetheless, one must consider that the habit of smoking was acquired by women at a later time^25^, and the delay in giving up that habit among women^26^ may be connected to the tendencies of stability and even increase of the disease in some anatomical locations. This fact may also explain that there is no difference in the birth cohorts of females, who have been adopting behaviors traditionally related to the male population^24^ ^,^ ^25^.

According to a 2019 report from the World Health Organization (WHO), smoking has diminished in most countries after the adoption of smoking control programs, guided by the MPOWER strategy^27^. These global evaluations place Brazil as one of the countries which stand out for this control^28^. These results are the effect of several regulatory measures adopted by the country, such as the ratification of the 2006 Framework Convention of the WHO on Tobacco Control^29^. Among the implemented measures are the monitoring of tobacco use; the raising of taxes on tobacco products; the prohibition of advertising of tobacco products; Brazilian Law number 12,546 from 2011, which created tobacco free public environments^30^; and Brazilian Decree number 8,262/2014, which regulated such environments and determined the increase in possible warnings^31^.

The increase in incidence and mortality for cancer with age, which was confirmed by the age-period-cohort model, shows the impact of the cumulative process of exposure to carcinogenic factors, especially exposure to smoking in the past, combined with population aging^32^. The same pattern had already been confirmed in the incidence and mortality rates of lip and oral cavity cancers, from 1990 to 2017,^33^ and in other international statistics^34^, as well as in an analysis of mortality from oral and oropharyngeal cancers in Brazil between 1983 and 2017^35^. Older individuals are also those with the least education and those which smoke the most, according to previous studies performed in Brazil^26^ ^,^ ^36^. For more recent cohorts, however, urbanization may have contributed to quitting the habit of smoking^26^. 

Male individuals also presented the highest mortality rates for cancers of all the anatomical regions and age groups. Perea et al.^10^ also reached the same result when analyzing the mortality rates of mouth and pharynx cancer in Brazil between 2010 and 2013. This study demonstrated that eight in every ten deaths occurred among men. This finding relates to the higher incidence rates also may well be related to the fact that men do not seek out medical care as commonly, or seek it out in later stages of the disease^37^ ^,^ ^38^. This pattern was also verified globally in several international studies^33^ ^,^ ^34^ ^,^ ^39^. In another study conducted in Brazil from 1983 and 2017, the mortality from oral and oropharyngeal cancer was assessed. In this analysis, the average death rate among men was much higher than among women^35^. Regarding lip cancer, a recent study with data from referral centers of the five Brazilian geographic regions demonstrated that the occurrence of lip squamous cell carcinoma is higher among men^21^. 

The trend of decline in mortality that was observed for all the cancers studied in this work might be a result of the increase in early detection of lesions in more recent years, enabling early diagnosis, improvements in treatment for these kinds of cancers, and a change in the patterns of alcohol consumption and smoking in recent years. Such considerations are confirmed by the reduction in confirmed risk, especially in more recent cohorts, from the last decade. A study conducted in Brazil indicated that the population has been seeking out medical care more often^40^. This scenario may have been influenced by the improvements in accessibility and quality of oral healthcare services^41^ ^,^ ^42^, since mortality for oral cancer is inversely proportional to availability and financing of primary care services^4^.

This behavior has been different for nasopharynx cancer, which has shown an increase in incidence and mortality rates. Cancers affecting this anatomical region differ from other tumors in the head and neck, as they present a closer relation to more aggressive EBV subtypes, stimulated by genetic factors and with a non-specific or non-present symptomatology, even in advanced stages, and because they are more difficult to treat effectively^43^.

A 20-year trend analysis conducted in Estonia also identified an increase in incidence of nasopharyngeal cancers and suggested that HPV infection may well be a contributing cause for those kinds of cancers^44^. In Thailand, head and neck cancers, as a group, are among the three more common cancers in men and the fifth in women. These higher incidences of nasopharynx cancer in Thailand and in the whole of southern Asia, in comparison to global data, have been related to local dietary habits, based on fish saturated with salt, and the role of the HPV has been poorly studied to date^45^. In Brazil, a systematic revision indicated a high prevalence of HPV infection, although the data is scarce for some regions, with variations according to the anatomical location and lower rates (11.9%) in oral cavity samples^46^. The prevalence of lesions of squamous cell carcinoma in the head and neck is estimated in 26%^47^. 

The results of the trend series updated to 2019 for Brazil showed a pattern that is different from that confirmed globally for the 1990-2017 period. The estimated global incidence has decreased dramatically for nasopharynx cancers and has increased for lip and oral cavity cancers^48^. In the meantime, specific analysis of lip and oral cavity cancers has indicated an increase in incidence and stability in mortality, but with great variability among regions^33^. It is important to emphasize that, when new data is included with each new cycle of the GBD, the entire historical series is updated and new methods are incorporated into the model^13^, which partially explains the differences in comparison with previous estimates. Although more developed countries have seen a reduction in smoking and an increase in HPV, many other countries have been experiencing growing or stable rates of head and neck cancers, still primarily related to smoking and alcohol consumption^8^. One study conducted in Central and South American countries, with data from population records, indicated Brazil as the country with the highest incidence rates of lip, oral cavity, and pharynx cancers for individuals from both sexes and stable trends for the period of 1997-2006^49^. According to data from the WHO, mortality has followed the same pattern^49^. A trend of decline was noted in Argentina and an increase in incidence in females has been confirmed in Chile. The prevalence of tumors related to HPV in Central and South America is low when compared to other regions. The authors emphasized that, regardless of the policies already implemented for tobacco and alcohol control, the rates are high due mainly to difficulties in the implementation of an efficient network for the diagnosis of mouth cancer. There is also no integration of primary, secondary, and tertiary oral healthcare services; a lack of knowledge and experience of primary care dentists in identifying malignant lesions or doing biopsies; and difficulties in reaching the at-risk population.

It is important to emphasize that the analytical methodology used by the GBD study had different stages of modeling and adjustment, resulting in estimates which are different from those observed directly in the records of the Mortality Information System (SIM), in terms of applying adjustments for the sub-registry and redistribution of garbage codes, and due to the use of such sources as vital registration and cancer registry data, as well as the use of standardized rates and other adjustments. Therefore, the inherent limitations of the methodology of the GBD study also apply to the present study. Moreover, the ICD change from version 9 to 10, in 1996, may have led to different results concerning the classification of the underlying cause of death, but this change is not expected to modify the trends described herein, as we have conducted an analysis that included the last 30 years. There was an improvement in data quality over the period: the completeness of death counts increased from 80%, in 1980-1991, to 95%, in 2000-2010^50^, but a large regional variation still persists in a pattern similar to the distribution of ill-defined causes of death^14^. A quality factor is that mouth and lip cancers are considered as a group, even though they have distinct risk factors and characteristics, which might have influenced the estimates shown in this study, especially in relation to incidence. However, it is important to emphasize that the GBD has been widely published and used by researchers and decision-makers for approaching different themes. Since the GBD studies use a standardized methodology, they allow for more comparability between places and periods for which the availability and quality of data are heterogeneous. 

The present study confirmed a trend of decline of incidence and mortality of lip, oral cavity and pharynx cancers over the last 30 years in the Brazilian adult population (30 years of age and older). The time series analysis showed different patterns for cancers, affecting some anatomical locations, especially in women. The highest incidence and mortality rates were of lip and oral cavity cancers in men. The age-period-cohort model converged with the results, indicating an increase in mortality, which progressed with age, decline in incidence rates and, consequently, mortality rates in the more recent cohorts, which is a reflection of the improved access to health services for early diagnosis and adequate treatment, which has been observed recently, especially in the last decade. 
