# Supplementary material for: Incidence and mortality rates of lip, oral cavity, and pharynx cancers in Brazil: time-trend and age-period-cohort analysis from the last 30 years, Global Burden of Disease Study
Source: Rev Soc Bras Med Trop. 2022 Jan 28;55(Suppl 1):e0286-2021. doi: 10.1590/0037-8682-0286-2021 (PMC9009427; doi:10.1590/0037-8682-0286-2021)
Supplement: Supplementary file 1 [file 1678-9849-rsbmt-55-s01-e0286-2021-supp1.pdf]

SUPPLEMENTARY TABLE 1: Standardized rates and AAPC of the incidence and mortality rates of lip, oral cavity, and pharynx cancers (per 100,000 inhabitants), according to anatomical location and sex. Brazil, 1990-2019.

Cause	Sex	Age group	1990	1991	1992	1993	1994	1995	1996	1997	1998	1999	2000	2001	2002	2003	2004	2005	2006	2007	2008	2009	2010	2011	2012	2013	2014	2015	2016	2017	2018	2019	AAPC (95%CI)
			Incidence																														
Lip and oral cavity cancer	Male	30-59	8.55	8.50	8.58	8.79	8.89	8.93	9.12	9.12	9.26	9.42	9.47	9.54	9.49	9.57	9.53	9.31	9.21	9.11	9.00	8.91	8.80	8.71	8.51	8.41	8.19	8.01	7.97	7.67	7.68	7.71	-0.3* (-0.5;-0.2)
		60+	28.50	28.30	28.54	29.42	29.40	29.24	29.45	29.27	29.59	30.09	30.18	30.26	30.52	30.97	31.02	30.26	30.01	29.73	29.54	29.31	29.28	29.30	29.00	28.86	28.83	28.98	29.39	28.81	28.32	28.35	0.0 (-0.2;0.3)
		Std 30+	13.83	13.74	13.86	14.25	14.32	14.30	14.51	14.45	14.64	14.89	14.95	15.02	15.06	15.23	15.22	14.86	14.72	14.57	14.44	14.31	14.22	14.16	13.94	13.82	13.65	13.56	13.64	13.27	13.14	13.17	-0.2* (-0.2;-0.1)
	Female	30-59	1.62	1.62	1.66	1.74	1.76	1.79	1.84	1.85	1.90	1.92	1.91	1.94	1.96	1.93	1.95	1.95	1.96	1.97	1.98	1.98	1.95	1.94	1.91	1.88	1.83	1.80	1.78	1.73	1.72	1.72	0.2* (0.1;0.3)
		60+	10.21	10.06	10.10	10.31	10.40	10.39	10.54	10.54	10.55	10.79	10.70	10.74	10.99	10.95	11.06	11.00	11.07	11.07	11.06	11.03	10.98	11.02	10.85	10.73	10.56	10.42	10.36	10.15	10.15	10.13	-0.0 (-0.1;0.1)
		Std 30+	3.89	3.85	3.90	4.01	4.05	4.06	4.14	4.15	4.19	4.27	4.24	4.27	4.35	4.32	4.36	4.35	4.37	4.38	4.38	4.37	4.34	4.35	4.27	4.22	4.14	4.08	4.05	3.96	3.95	3.95	0.1 (-0.0;0.2)
Nasopharynx cancer	Male	30-59	0.58	0.59	0.61	0.64	0.65	0.69	0.72	0.73	0.77	0.78	0.79	0.80	0.80	0.80	0.79	0.79	0.79	0.78	0.77	0.77	0.76	0.75	0.74	0.72	0.69	0.68	0.68	0.67	0.67	0.68	0.6* (0.3;0.9)
		60+	0.81	0.82	0.85	0.87	0.92	0.96	1.01	1.05	1.08	1.14	1.16	1.17	1.21	1.21	1.20	1.19	1.17	1.15	1.13	1.10	1.09	1.07	1.06	1.05	1.03	1.05	1.03	1.02	1.00	1.00	0.7* (0.5;1.0)
		Std 30+	0.64	0.65	0.67	0.70	0.72	0.76	0.80	0.82	0.85	0.88	0.89	0.90	0.91	0.91	0.90	0.89	0.89	0.88	0.87	0.86	0.85	0.84	0.82	0.81	0.78	0.78	0.78	0.76	0.76	0.76	0.6* (0.3;0.9)
	Female	30-59	0.18	0.18	0.18	0.19	0.19	0.20	0.21	0.22	0.22	0.23	0.23	0.24	0.25	0.25	0.26	0.25	0.25	0.24	0.24	0.24	0.24	0.24	0.24	0.23	0.23	0.22	0.23	0.22	0.22	0.22	0.8* (0.6;0.9)
		60+	0.41	0.41	0.41	0.45	0.43	0.45	0.46	0.48	0.48	0.52	0.52	0.50	0.54	0.54	0.54	0.51	0.51	0.52	0.51	0.50	0.50	0.50	0.49	0.49	0.48	0.46	0.46	0.45	0.44	0.44	0.3* (0.1;0.5)
		Std 30+	0.24	0.24	0.24	0.26	0.25	0.26	0.27	0.29	0.29	0.31	0.31	0.31	0.32	0.32	0.33	0.32	0.32	0.32	0.31	0.31	0.31	0.31	0.30	0.30	0.30	0.28	0.29	0.28	0.28	0.28	0.6* (0.4;0.8)
Other pharynx cancer	Male	30-59	4.54	4.55	4.63	4.75	4.78	4.81	4.92	4.97	5.13	5.26	5.33	5.35	5.43	5.45	5.45	5.38	5.30	5.23	5.17	5.14	5.09	5.03	4.90	4.82	4.70	4.61	4.64	4.55	4.58	4.63	0.1 (-0.2;0.3)
		60+	14.33	14.28	14.54	15.04	15.04	15.19	15.47	15.37	15.48	15.69	15.89	15.88	16.01	16.16	16.28	16.04	15.84	15.67	15.62	15.47	15.48	15.36	15.10	14.95	14.85	14.92	15.19	14.95	14.90	15.02	0.1* (0.0;0.3)
		Std 30+	7.13	7.13	7.25	7.47	7.50	7.55	7.71	7.72	7.87	8.02	8.13	8.13	8.23	8.29	8.32	8.20	8.09	7.99	7.94	7.87	7.84	7.77	7.60	7.50	7.39	7.34	7.43	7.30	7.31	7.38	0.1* (0.0;0.2)
	Female	30-59	0.59	0.59	0.60	0.62	0.61	0.63	0.63	0.63	0.64	0.66	0.66	0.67	0.68	0.67	0.68	0.67	0.67	0.67	0.67	0.67	0.66	0.67	0.66	0.63	0.63	0.62	0.62	0.60	0.60	0.60	0.0 (-0.1;0.2)
		60+	2.90	2.83	2.82	2.86	2.83	2.80	2.77	2.67	2.67	2.71	2.73	2.66	2.73	2.72	2.74	2.76	2.79	2.77	2.75	2.74	2.74	2.73	2.69	2.63	2.59	2.56	2.58	2.56	2.57	2.57	-0.4* (-0.7;-0.0)
		Std 30+	1.20	1.18	1.19	1.21	1.20	1.20	1.20	1.17	1.18	1.20	1.21	1.20	1.22	1.21	1.23	1.22	1.23	1.22	1.22	1.22	1.21	1.21	1.19	1.16	1.15	1.13	1.14	1.12	1.12	1.12	-0.2* (-0.4;-0.0)
Head and Neck cancer	Male	30-59	13.68	13.63	13.81	14.16	14.32	14.42	14.76	14.83	15.16	15.47	15.58	15.68	15.72	15.83	15.78	15.49	15.31	15.12	14.94	14.81	14.65	14.49	14.15	13.96	13.59	13.30	13.29	12.88	12.93	13.01	-0.1 (-0.4;0.1)
		60+	43.65	43.44	43.87	45.36	45.39	45.37	45.93	45.68	46.15	46.92	47.25	47.30	47.73	48.34	48.50	47.48	47.00	46.54	46.30	45.90	45.84	45.73	45.16	44.86	44.72	44.96	45.63	44.78	44.20	44.36	0.1 (-0.2;0.3)
		Std 30+	21.61	21.52	21.77	22.42	22.54	22.61	23.01	22.99	23.36	23.80	23.96	24.05	24.20	24.43	24.44	23.96	23.70	23.43	23.24	23.04	22.90	22.76	22.36	22.14	21.83	21.68	21.85	21.32	21.21	21.31	-0.1* (-0.2;-0.0)
	Female	30-59	2.39	2.39	2.44	2.53	2.59	2.62	2.69	2.69	2.77	2.81	2.81	2.85	2.88	2.84	2.89	2.87	2.88	2.87	2.90	2.90	2.85	2.85	2.81	2.75	2.69	2.64	2.63	2.54	2.54	2.54	0.2* (0.1;0.3)
		60+	13.51	13.27	13.33	13.59	13.65	13.60	13.80	13.70	13.73	13.98	13.96	13.92	14.25	14.17	14.35	14.27	14.38	14.35	14.33	14.28	14.21	14.25	14.02	13.84	13.62	13.44	13.39	13.18	13.16	13.14	-0.1* (-0.1;-0.0)
		Std 30+	5.33	5.27	5.32	5.46	5.51	5.53	5.63	5.60	5.67	5.77	5.76	5.78	5.89	5.84	5.92	5.89	5.92	5.91	5.92	5.91	5.86	5.87	5.77	5.68	5.58	5.50	5.48	5.36	5.35	5.34	0.0 (-0.1;0.1)
			Mortality																														
Lip and oral cavity cancer	Male	30-59	4.81	4.76	4.78	4.87	4.90	4.90	4.99	4.96	5.03	5.09	5.10	5.12	5.08	5.10	5.06	4.94	4.87	4.80	4.74	4.69	4.60	4.53	4.41	4.35	4.22	4.13	4.10	3.93	3.91	3.90	-0.7* (-0.8;-0.5)
		60+	20.76	20.48	20.48	20.96	20.87	20.58	20.65	20.46	20.60	20.93	20.96	20.97	21.10	21.40	21.39	20.81	20.64	20.44	20.30	20.13	20.02	19.92	19.63	19.46	19.41	19.52	19.72	19.23	18.82	18.76	-0.4* (-0.6;-0.1)
		Std 30+	9.03	8.92	8.94	9.13	9.13	9.05	9.14	9.07	9.15	9.28	9.30	9.32	9.32	9.41	9.39	9.14	9.04	8.94	8.86	8.77	8.68	8.60	8.44	8.35	8.24	8.20	8.24	7.98	7.85	7.84	-0.5* (-0.5;-0.4)
	Female	30-59	0.83	0.81	0.84	0.86	0.87	0.87	0.89	0.88	0.91	0.91	0.89	0.90	0.90	0.88	0.89	0.88	0.88	0.88	0.88	0.88	0.86	0.85	0.83	0.81	0.79	0.77	0.76	0.73	0.73	0.73	-0.5* (-0.6;-0.3)
		60+	7.92	7.74	7.68	7.79	7.79	7.71	7.82	7.73	7.68	7.83	7.72	7.72	7.87	7.78	7.84	7.78	7.80	7.80	7.79	7.75	7.69	7.69	7.55	7.44	7.34	7.21	7.17	7.01	6.98	6.93	-0.4* (-0.5;-0.3)
		Std 30+	2.70	2.65	2.65	2.69	2.70	2.68	2.72	2.70	2.70	2.74	2.70	2.71	2.75	2.71	2.73	2.71	2.71	2.71	2.71	2.70	2.67	2.66	2.61	2.57	2.52	2.48	2.46	2.40	2.38	2.37	-0.5* (-0.5;-0.4)
Nasopharynx cancer	Male	30-59	0.52	0.53	0.53	0.56	0.57	0.61	0.62	0.64	0.66	0.67	0.68	0.68	0.69	0.67	0.66	0.65	0.65	0.64	0.63	0.62	0.62	0.60	0.59	0.57	0.55	0.53	0.54	0.52	0.52	0.52	-0.0 (-0.4;0.3)
		60+	0.86	0.91	0.88	0.96	0.98	1.03	1.11	1.11	1.16	1.21	1.22	1.25	1.28	1.27	1.24	1.25	1.22	1.20	1.17	1.16	1.13	1.12	1.10	1.08	1.06	1.08	1.07	1.04	1.03	1.01	0.6* (0.4;0.7)
		Std 30+	0.61	0.63	0.63	0.67	0.68	0.72	0.75	0.77	0.79	0.81	0.82	0.83	0.84	0.83	0.81	0.81	0.80	0.79	0.77	0.76	0.75	0.74	0.72	0.71	0.69	0.68	0.68	0.66	0.65	0.65	0.2* (0.0;0.5)
	Female	30-59	0.15	0.15	0.15	0.16	0.16	0.16	0.17	0.17	0.18	0.18	0.19	0.19	0.19	0.19	0.19	0.19	0.18	0.19	0.18	0.18	0.18	0.17	0.17	0.16	0.16	0.15	0.16	0.15	0.14	0.14	-0.1 (-0.3;0.1)
		60+	0.44	0.46	0.44	0.47	0.48	0.48	0.49	0.52	0.51	0.54	0.54	0.55	0.56	0.55	0.56	0.55	0.54	0.53	0.52	0.52	0.52	0.51	0.49	0.50	0.48	0.47	0.47	0.45	0.44	0.45	0.0 (-0.1;0.2)
		Std 30+	0.23	0.23	0.23	0.24	0.25	0.25	0.26	0.26	0.27	0.28	0.28	0.29	0.29	0.29	0.29	0.28	0.28	0.28	0.27	0.27	0.27	0.26	0.25	0.25	0.24	0.24	0.24	0.23	0.22	0.22	-0.1 (-0.2;0.1)
Other pharynx cancer	Male	30-59	3.91	3.90	3.95	4.04	4.05	4.05	4.12	4.15	4.26	4.35	4.38	4.36	4.40	4.40	4.37	4.29	4.21	4.13	4.07	4.03	3.97	3.90	3.77	3.69	3.59	3.51	3.53	3.44	3.44	3.45	-0.4* (-0.7;-0.1)
		60+	14.73	14.63	14.83	15.29	15.21	15.32	15.53	15.39	15.43	15.56	15.77	15.67	15.79	15.84	15.90	15.62	15.35	15.18	15.10	14.90	14.85	14.66	14.36	14.18	14.05	14.09	14.31	14.01	13.89	13.93	-0.2* (-0.3;-0.1)
		Std 30+	6.77	6.74	6.83	7.02	7.00	7.03	7.14	7.12	7.21	7.32	7.39	7.36	7.42	7.43	7.42	7.29	7.16	7.05	6.99	6.90	6.85	6.75	6.57	6.47	6.36	6.31	6.38	6.24	6.20	6.23	-0.3* (-0.4;-0.2)
	Female	30-59	0.47	0.48	0.48	0.48	0.49	0.49	0.49	0.47	0.49	0.49	0.49	0.49	0.49	0.48	0.48	0.47	0.47	0.46	0.46	0.46	0.45	0.44	0.44	0.41	0.41	0.40	0.40	0.39	0.38	0.38	-0.8* (-1.0;-0.6)
		60+	3.01	2.92	2.87	2.92	2.87	2.81	2.79	2.67	2.66	2.64	2.66	2.58	2.63	2.61	2.62	2.63	2.64	2.61	2.59	2.58	2.55	2.53	2.48	2.42	2.38	2.37	2.36	2.33	2.32	2.31	-0.9* (-1.1;-0.7)
		Std 30+	1.15	1.12	1.11	1.13	1.12	1.10	1.10	1.05	1.06	1.06	1.06	1.05	1.05	1.04	1.05	1.04	1.04	1.03	1.02	1.02	1.00	1.00	0.98	0.94	0.93	0.92	0.92	0.90	0.89	0.89	-0.9* (-1.1;-0.6)
Head and Neck cancer	Male	30-59	9.25	9.19	9.27	9.47	9.53	9.55	9.74	9.75	9.94	10.11	10.15	10.17	10.16	10.17	10.09	9.88	9.73	9.57	9.44	9.33	9.19	9.03	8.77	8.62	8.37	8.18	8.16	7.89	7.87	7.88	-0.5* (-0.7;-0.4)
		60+	36.34	35.98	36.20	37.22	37.08	36.95	37.29	36.93	37.19	37.70	37.94	37.88	38.13	38.49	38.55	37.69	37.24	36.82	36.59	36.19	36.01	35.68	35.10	34.72	34.53	34.66	35.10	34.30	33.73	33.71	-0.3* (-0.3;-0.2)
		Std 30+	16.42	16.28	16.40	16.82	16.82	16.81	17.03	16.95	17.16	17.41	17.50	17.51	17.56	17.67	17.62	17.24	17.01	16.78	16.63	16.44	16.29	16.09	15.74	15.52	15.29	15.19	15.29	14.88	14.71	14.71	-0.4* (-0.5;-0.3)
	Female	30-59	1.46	1.44	1.46	1.50	1.51	1.52	1.55	1.53	1.57	1.58	1.57	1.58	1.58	1.55	1.56	1.54	1.53	1.52	1.52	1.51	1.48	1.47	1.43	1.39	1.36	1.33	1.32	1.27	1.25	1.25	-0.5* (-0.7;-0.4)
		60+	11.36	11.10	11.05	11.17	11.13	11.00	11.09	10.91	10.85	11.00	10.91	10.85	11.06	10.94	11.03	10.94	10.97	10.94	10.90	10.85	10.77	10.73	10.52	10.36	10.20	10.05	9.99	9.79	9.75	9.69	-0.5* (-0.6;-0.4)
		Std 30+	4.08	4.00	4.00	4.06	4.06	4.03	4.07	4.01	4.03	4.08	4.04	4.04	4.09	4.04	4.07	4.03	4.03	4.01	4.00	3.98	3.94	3.92	3.84	3.77	3.70	3.64	3.61	3.53	3.50	3.48	-0.5* (-0.6;-0.5)

AAPC = annual average percent change
CI= Confidence Interval
“Std” (Standardized)
*Statistical significance at level of 5%
