# Supplementary material for: Incidence and mortality rates of lip, oral cavity, and pharynx cancers in Brazil: time-trend and age-period-cohort analysis from the last 30 years, Global Burden of Disease Study
Source: Rev Soc Bras Med Trop. 2022 Jan 28;55(Suppl 1):e0286-2021. doi: 10.1590/0037-8682-0286-2021 (PMC9009427; doi:10.1590/0037-8682-0286-2021)
Supplement: Supplementary file 2 [file 1678-9849-rsbmt-55-s01-e0286-2021-supp2.pdf]

SUPPLEMENTARY TABLE 2: Age-Period-Cohort model of the incidence and mortality rates of lip, oral cavity, and pharynx cancers, according to anatomical location and sex. Brazil, 1990-2019.

Model	Incidence								Mortality							
	**Male**				**Female**				**Male**				**Female**			
	**df**	**Deviance**	**Deviance test**	**p-value**	**df**	**Deviance**	**Deviance test**	**p-value**	**df**	**Deviance**	**Deviance test**	**p-value**	**df**	**Deviance**	**Deviance test**	**p-value**
Lip and oral cavity cancer																
Age	61	864.2			61	139.8			61	1027.0			61	176.3		
Age-drift	60	728.3	135.9	<0.001	60	139.7	0.1	0.799	60	784.0	243.0	<0.001	60	134.2	42.1	<0.001
Age-Cohort	57	359.9	368.4	<0.001	57	137.0	2.7	0.440	57	518.0	266.0	<0.001	57	122.4	11.8	0.008
Age-Period-Cohort	54	248.1	111.8	**<0.001**	54	62.4	74.7	**<0.001**	54	469.9	48.2	**<0.001**	54	100.9	21.5	**<0.001**
Age-Period	57	537.8	289.7	<0.001	57	63.3	1.0	0.809	57	691.0	221.1	<0.001	57	107.3	6.4	<0.001
Age-drift	60	728.3	190.5	<0.001	60	139.7	76.4	<0.001	60	784.0	93.0	<0.001	60	134.2	26.8	<0.001
Nasopharynx cancer																
Age	61	200.0			61	48.9			61	135.3			61	40.9		
Age-drift	60	199.6	0.4	<0.001	60	47.2	1.7	0.194	60	128.9	6.4	0.012	60	37.9	3.0	0.086
Age-Cohort	57	196.0	3.6	<0.001	57	46.3	0.9	0.826	57	113.4	15.5	0.001	57	36.3	1.6	0.654
Age-Period-Cohort	54	136.5	59.5	**<0.001**	54	22.5	23.8	<0.001	54	66.4	46.9	**<0.001**	54	19.5	16.8	**0.001**
Age-Period	57	136.6	0.1	<0.001	57	25.5	3.1	0.379	57	72.2	5.7	0.125	57	19.7	0.3	0.968
Age-drift	60	199.6	63.0	<0.001	60	47.2	21.6	**<0.001**	60	128.9	56.7	<0.001	60	37.9	18.2	<0.001
Other pharynx cancer																
Age	61	720.9			61	56.8			61	908.6			61	137.2		
Age-drift	60	704.6	16.3	<0.001	60	51.8	5.0	**0.025**	60	747.5	161.1	<0.001	60	76.7	60.5	**<0.001**
Age-Cohort	57	537.4	167.1	<0.001	57	48.8	3.0	0.389	57	561.8	185.7	<0.001	57	69.0	7.6	0.055
Age-Period-Cohort	54	426.9	110.6	**<0.001**	54	40.5	8.3	0.041	54	479.0	82.8	**<0.001**	54	66.9	2.1	0.553
Age-Period	57	550.0	123.1	<0.001	57	42.6	2.1	0.551	57	622.5	143.5	<0.001	57	72.8	5.8	0.120
Age-drift	60	704.6	154.6	<0.001	60	51.8	9.2	0.027	60	747.5	125.0	<0.001	60	76.7	3.9	0.273
Head and Neck cancer																
Age	61	1657.5			61	157.7			61	1845.1			61	282.6		
Age-drift	60	1526.3	131.2	<0.001	60	156.8	0.9	0.348	60	1439.5	405.5	<0.001	60	188.4	94.2	<0.001
Age-Cohort	57	979.3	547.0	<0.001	57	153.5	3.3	0.344	57	995.5	444.0	<0.001	57	168.0	20.4	<0.001
Age-Period-Cohort	54	724.1	255.2	**<0.001**	54	59.3	94.2	**<0.001**	54	839.6	155.9	**<0.001**	54	139.6	28.4	**<0.001**
Age-Period	57	1138.2	414.1	<0.001	57	59.5	0.3	0.965	57	1186.3	346.7	<0.001	57	150.0	10.4	0.015
Age-drift	60	1526.3	388.1	<0.001	60	156.8	97.3	<0.001	60	1439.5	253.2	<0.001	60	188.4	38.4	<0.001

df: degrees of freedom
